# *cnnm-5* knockdown improves proteostasis of mutant Huntingtin protein in *C. elegans*

**DOI:** 10.17912/micropub.biology.001497

**Published:** 2025-08-01

**Authors:** Matthew Hull, Joslyn Mills

**Affiliations:** 1 Biology, Bridgewater State University, Bridgewater, Massachusetts, United States; 2 Biology, Wheaton College - Massachusetts, Norton, Massachusetts, United States

## Abstract

Huntington's disease (HD) is an age-related neurodegenerative disease associated with the aggregation of mutant Huntingtin protein (mHTT). It is theorized that prevention or clearance of these aggregates through autophagy and the ubiquitin proteasome system (UPS) protects neurons from degeneration. Using a *C. elegans* model of HD, a small reverse genetic screen of 100 random genes on Chromosome 3 identified *cnnm-5* as a genetic modifier of mHTT accumulation. During development, loss of *cnnm-5* by RNAi ( *cnnm-5* i) protects against mHTT accumulation, implicating *cnnm-5* as a negative regulator of protein aggregation prevention or clearance. Here we report that knocking down *cnnm-5* leads to decreased mHTT protein aggregation through the upregulation of the UPS and autophagy pathways, leading to increased lifespan. Further experimentation using a nematode model of Alzheimer's disease demonstrates *cnnm-5* i protects against paralysis by decreasing beta amyloid protein misfolding in body wall muscles.

**Figure 1. Modulation of cnnm-5 affects protein aggregation, proteostasis, lifespan, and paralysis of C. elegans:**
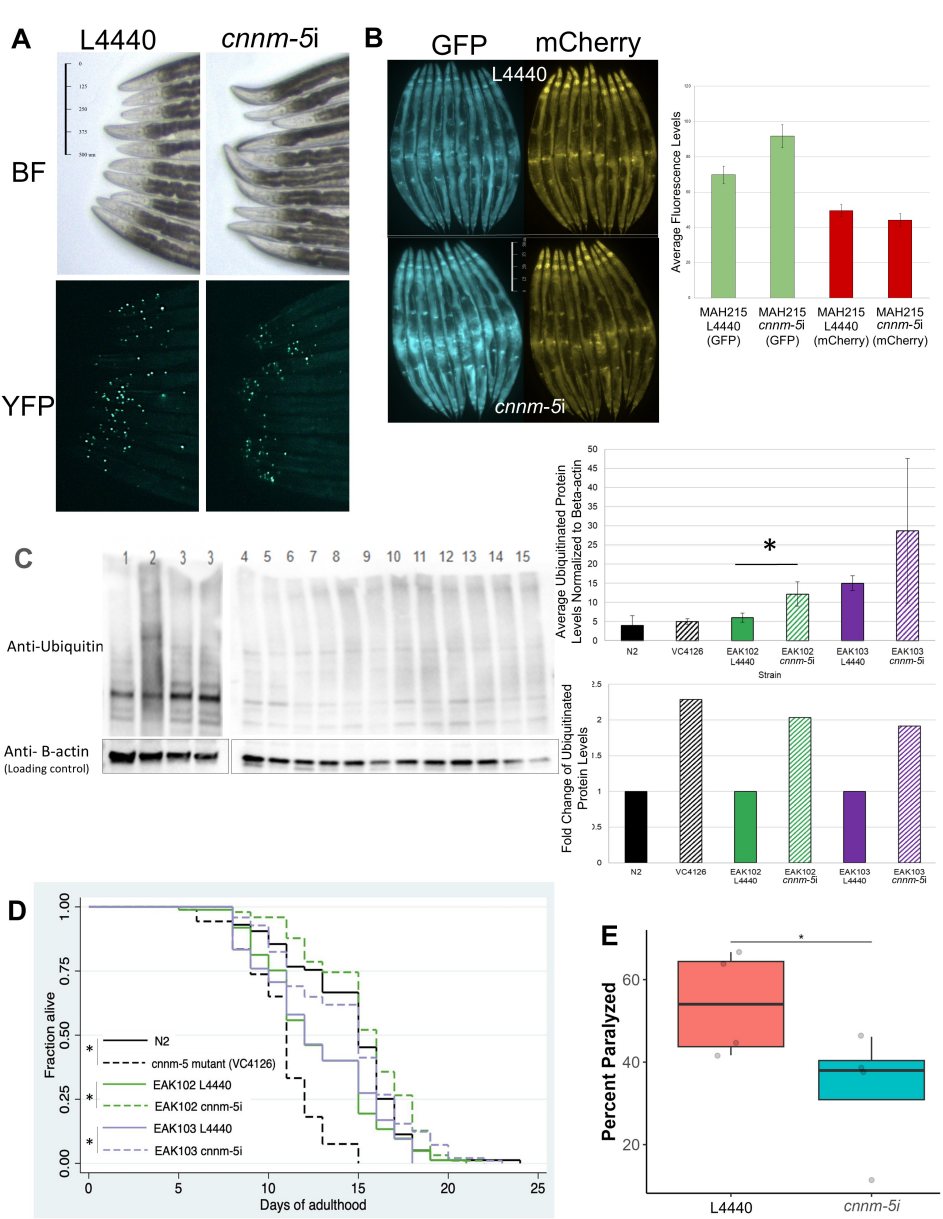
**A)** Day 1 EAK103 adults developed on L4440 (control) or *cnnm-5* i at 25°C, zoomed in on the head region. Puncta indicate accumulation of mHTT tagged with YFP. Fluorescent images were taken using the GFP filter on the stereoscope at 63x. Scale bar indicates 500μm. **B)** MAH215 nematodes were imaged on Day 1 of adulthood after development at 25°C on L4440 (control) or *cnnm-5* i. The green channel shows autophagosomes only (AP), and the red channel shows both autophagosomes and autolysosomes (AP + AL). Average autophagy levels in MAH215 L4440 or *cnnm-5* i were quantified using ImageJ, p<0.05. Images in A & B are representative of three trials. **C)** Levels of ubiquitinated proteins in WT (lanes 1 & 2-biological replicates) and VC4126 (triple mutant strain that includes a nonsense mutation in *cnnm-5* ) (lanes 3 & 3, technical replicates) on OP50 and EAK102 and EAK103 on control (L4440) ( EAK102 lanes 4-6, EAK103 lanes 10-12, biological triplicates) or *cnnm-5* i ( EAK102 lanes 7-9, EAK103 lanes 13-15, biological triplicates). Beta-actin is shown as the loading control. The average ubiquitin signal was quantified and normalized to beta-actin. Two-tailed Student's *t* -test was performed to determine p value: N2 vs VC4126 p=0.65; EAK102 L4440 vs *cnnm-5* i p=0.03 (*significant); EAK103 L4440 vs *cnnm-5* i p=0.27. Fold change of normalized ubiquitin signal is based on comparison to the appropriate non- *cnnm-5* -modified control (color matched). **D)** Survival curve of nematodes that survived each day of adulthood at 25°C. N2 and VC4126 were grown on OP50 , and EAK102 and EAK103 were developed and maintained on control (L4440) or *cnnm-5* i. n=100 worms per condition. A chi-square test was performed to determine significance, *p<0.05. **E)** Percent of GMC101 nematodes that were paralyzed at adulthood Day 4 after 72 hours under heat stress (25°C) developed and grown on control (L4440) or *cnnm-5* i. Each dot represents a separate trial (n>20 per trial). Bar within box represents the mean of all trials, and the whiskers represent standard error. One-tailed Student's *t* -test was performed to determine significance, *p=0.042.

## Description

Huntington's disease (HD) is a monogenic autosomal dominant disease caused by a mutation in the gene that codes for huntingtin protein (HTT). The mutated HTT (mHTT) expresses excessive glutamine repeats (polyQ), causing the mutant protein to aggregate, with a positive correlation between aggregation propensity and number of polyQ repeats (Finkbeiner, 2011). These proteins are believed to aggregate through the evasion of protein homeostasis, or proteostasis, which is accomplished by clearing damaged or aggregated proteins. This clearing of proteins can be carried out by two main pathways: autophagy and the ubiquitin proteasome system (UPS). Macroautophagy, further referred to as autophagy, clears proteins and other macromolecules in bulk by degrading and recycling in an autophagolysosome-dependent manner, while the UPS targets individual proteins tagged with ubiquitin to be degraded by the proteasome. The goal of proteostasis is to clear proteins to keep the cell healthy; therefore, a decrease in functional proteostasis could be driving the Huntingtin protein aggregates to accumulate, progressing the disease. Here we use *Caenorhabditis elegans* to begin to elucidate the genetic players in this theory.

*C. elegans* are microscopic nematodes that have been used extensively in biomedical research due to their short life cycle and lifespan, genetic homology to humans, and simple genetic manipulation (Riddle et al., 1997). A number of *C. elegans* models for HD have been developed, and this study used the EAK102 and EAK103 strains to investigate genetic modifiers of protein aggregation. These animals have been engineered to include a 513 amino acid fragment sequence of human HTT that is expressed in the body wall muscles of *C. elegans .* EAK102 expresses HTT with the normal 15 polyQ repeats while EAK103 expresses mHTT with 128 polyQ repeats. Both EAK102 and EAK103 express the HTT in the body wall muscles tagged with YFP, specifically to highlight the muscle dysfunctions caused by aggregation-prone proteins. EAK103 reveals the toxic effects of the increased polyQ repeats associated with HD by causing paralysis due to the accumulation of mHTT aggregates in the muscle cells (Lee et al., 2017). Using EAK103 in a small general reverse genetic screen with RNAi targeting 100 genes located on the *C. elegans '* Chromosome 3 based on a randomly selected plate of RNAi from the Ahringer Library (Qu et al., 2011), the gene *cnnm-5* was qualitatively identified as a genetic modifier of the mHTT aggregation, revealing a decrease in the number of mHTT aggregates as well as decreased fluorescence when *cnnm-5* was knocked down through development ( Figure 1A ).

*cnnm-5* is a member of the *cnnm* gene family ( *cnnm1-5* ), which are predicted to code for magnesium transport proteins that expel intracellular magnesium (Ishii et al., 2016). Magnesium supplementation in mice has been suggested to stimulate autophagy (Chen, 2023), though it is unknown the direct effect it has in *C. elegans* . Magnesium transport has been shown to support neuronal and muscle health through observing the effects that occur when magnesium pathways are disrupted, such as in osteoporosis, impaired brain development, and sperm motility (Giménez-Mascarell et al., 2019). Since the accumulation of mHTT aggregates appeared to decrease with the knockdown of *cnnm-5* , it could suggest knocking down this gene could be protecting the organism from a hallmark of Huntington's disease. *cnnm-5* , and possibly magnesium transport, may be negative regulators of protein aggregation clearance. If *cnnm-5* negatively regulates proteostatic machinery, then knockdown of *cnnm-5* is predicted to increase autophagy and the UPS, causing the decrease in mutant Huntingtin protein accumulation.

Normal functioning HTT plays a role in autophagy, including recognizing ubiquitin to load proteins that need to be degraded into the autophagosome (AP) and the subsequent transportation of APs (Saudou & Humbert, 2016). Expression of mHTT in mammalian HD models induces autophagy, due to the aggregation-prone proteins inactivating the mTOR pathway (Ravikumar et al., 2004), leading to elevated production of APs. However, there is a reduction in the AP function, reducing the amount of cargo loaded to be degraded (Saudou & Humbert, 2016). Elucidating the interactions between the proteins and the autophagosomal machinery would provide insight into how to repair this mechanism. To investigate if *cnnm-5* has any regulatory factors in the autophagy pathway of proteostasis, the MAH215 autophagy model strain was used in order to measure fluorescence levels indicating an upregulation or downregulation of autophagy. When *cnnm-5* is knocked down in MAH215 , there is an increase in fluorescence in GFP when compared to L4440, but no significant change in mCherry ( Figure 1B ). This increase in just GFP fluorescence indicates either that the autophagy pathway is being upregulated when *cnnm-5* is knocked down, or that the proteostatic machinery is being blocked, preventing the clearance of proteins. However, the decrease in mHTT aggregation observed when *cnnm-5* is knocked down ( Figure 1A ) supports the former, suggesting the mHTT aggregation in the control is occurring through an evasion of autophagy.

To determine if the proteasome was involved in mHTT clearance, the UPS pathway was independently evaluated using Western Blot analysis and quantification to test for ubiquitinated protein levels. When quantified, there were increased ubiquitinated protein levels when *cnnm-5* is knocked down ( Figure 1C ). Increased ubiquitin levels indicate that *cnnm-5* has a regulatory role in the UPS pathway of proteostasis. Since it is shown that *cnnm-5* knockdown decreased mHTT aggregation ( Figure 1A ), it is likely that the increased ubiquitin levels could be a result from proteostasis being upregulated rather than the proteostatic pathway being blocked, due to more proteins being ubiquitinated and labeled for degradation when *cnnm-5* is mutated or knocked down. Interestingly, there is also an increase of ubiquitinated protein levels in the EAK102 strain ( Figure 1C ) without the burden of mHTT protein. Deeper investigation is needed to determine the physiological and biochemical effects of mHTT with an intermediate number of polyQ repeats, or propensity to aggregate, in the context of *cnnm-5* i and its potential direct effect on the proteasome.

To test if the decreased mHTT aggregation upon *cnnm-5* knockdown would improve longevity of the EAK102 or EAK103 *C. elegans ,* a lifespan analysis was conducted. The VC4126 strain was also used to determine if a complete knockout of the gene had the same effect compared to the knockdowns and was compared to the Bristol N2 wildtype strain. When *cnnm-5* was knocked down in both the EAK102 and EAK103 strains, the lifespan of the *C. elegans* significantly increased compared to the no knockdown control L4440 ( Figure 1D ). With proteostasis being crucial to an organism's survival and health, the upregulation of autophagy and the UPS caused by knocking down *cnnm-5* improves the longevity of the *C. elegans* that express both HTT and mHTT. However, the genetic knockout, VC4126 , significantly decreased the lifespan of the *C. elegans* compared to the N2 wildtype. This decrease in longevity in the knockout could indicate that residual levels of *cnnm-5* in the knockdown experiments, particularly in the neurons that are refractory to RNAi, may play an important role in prolonging their lifespan.

Finally, to determine if *cnnm-5* knockdown was able to protect against a different protein aggregation-associated disease theorized to evade proteostatic machinery, a paralysis assay was conducted on the GMC101 Alzheimer's disease model strain. In this strain, the *C. elegans* become paralyzed due to the aggregation of beta amyloid in the body wall muscles (McColl et al., 2012). When *cnnm-5* was knocked down, the *C. elegans* were significantly protected from paralysis when compared to L4440 ( Figure 1E ). This indicates that the upregulation of proteostatic machinery that occurs from *cnnm-5* knockdown is protective in other aggregation-prone protein diseases.

Overall, the knockdown of *cnnm-5* appears to upregulate proteostasis to the point that diseases that persist with the evasion of proteostatic machinery are unable to do so as efficiently, decreasing their detrimental effects. This further indicates that progression of aggregation-associated neurodegenerative diseases relies on the evasion of proteostatic machinery, and research into genes that are regulators of proteostasis, such as *cnnm-5* , must be conducted to further discover interactions that decrease the mutant protein aggregates.

## Methods

***C.* *elegans* Handling & Imaging:** All worm handling was consistent across experiments unless otherwise specified. To synchronize the population starting at the egg stage, gravid adult *C.* *elegans* were bleached (Porta-de-la-Riva et al., 2012) to reduce variables caused by age. In knockdown experiments, *C.* *elegans* were fed *E. coli* ( HT115 ) that expresses RNAi targeting *cnnm-5* at 25°C through development unless otherwise noted. *E. coli* ( HT115 ) expressing only the L4440 plasmid backbone was used as the empty-vector non-targeting control. Fluorescent imaging was done by paralyzing the *C. elegans* with sodium azide, aligning the heads, and photographed using a TriTech epifluorescent stereoscope and iCapture Imaging software. All images are representative of multiple trials. The parameters for all comparative images were kept consistent across experiments (exposure, gain, and magnification). Images were analyzed using ImageJ software (version 1.8.0).

**Ubiquitinated Protein Analysis:** Total protein was collected from Day 1 adult EAK102 or EAK103 *C. elegans* developed on RNAi or N2 (WT) or VC4126 (triple mutant strain with nonsense mutation of interest in *cnnm-5* ) on OP50 at 25°C. Protein concentration was quantified by BCA (BioRad). 20 μg of total protein was loaded into each well of a 4-12% Tris-glycine gel and SDS-PAGE was performed. A Western Blot using anti-ubiquitin was performed to measure ubiquitinated protein levels in control (L4440) versus *cnnm-5* knockdown samples or N2 versus VC4126 . Stripped membranes were probed with anti-beta-actin and used as a loading control. **Lifespan Assay:** Age-synchronized *C. elegans* were developed at 20°C on OP50 ( N2 & VC4126 ) or at 25°C on *E. coli* HT115 expressing RNAi ( EAK102 & EAK103 ), transferred to 25°C on Day 1 of adulthood, and naturally dead worms were scored and removed nearly daily until 100% dead. Adult worms were transferred away from their progeny until post-fecundity. Death by means other than age were censored out of the analyzed population. Lifespan Assays were run concurrently between N2 , VC4126 , and EAK102 or EAK103 on empty-vector control (L4440) or *cnnm-5* i. n = 100 for each condition, with each condition run for three trials.

**Paralysis Assay:** Age-synchronized GMC101 *C. elegans* were developed at 20°C on *E. coli* HT115 expressing RNAi (+/- *cnnm-5* i). Naturally paralyzed worms were scored on day 4 of adulthood after 72 hours of heat stress (25°C). Paralysis assays were run concurrently between GMC101 on empty-vector control (L4440) or *cnnm-5* i. n>20 per condition per trial.

**Statistics** : Graphs were generated in Excel, Stata (version 16.1), or R (version 4.3.3) Statistical tests were performed in R (Student's *t* -test) or Stata (chi-squared test.)

## Reagents

**Normal growth media (NGM) plates:** Autoclave 20g agar, 3g NaCl, and 2.5g Bacto peptone in up to 1 liter of double distilled water, add 1.0 M potassium phosphate (pH 6.0), 1.0 M CaCl _2_ , 1.0 M MgSO _4_ , and 1.0 ml 5.0 mg/ml cholesterol in ethanol when cooled to 60C

**NGM + carbenicillin plates:** NGM recipe plus 1 ml of carbenicillin (100 mg/ml)

***E. coli* ( OP50 and HT115 strains) and RNAi library:** Ahringer RNAi library, Horizon Catalog #RCE1181

**M9 Buffer:** 35mM Sodium Phosphate Dibasic (Na _2_ HPO _4_ ), 0.103M Sodium Chloride, 22mM Potassium Phosphate Monobasic (KH _2_ PO _4_ ) and add 1mM MgSO _4_ after sterilization.

**Worm bleach:** 0.22M Sodium Hypochlorite and 0.786M Potassium Hydroxide in Millipore-filtered water

**Protein Quantification Assay Reagents (DC BCA):** Bio Rad Catalog #5000112

**Ubiquitin antibody:** Proteintech Catalog #10201-2-AP

**Beta-actin antibody:** Proteintech Catalog #81115-1-RR

**WesternSure Goat anti-rabbit HRP secondary:** Licor Catalog #926-80011

**SuperSignal™ West Femto Maximum Sensitivity Substrate:** Fisher Catalog #34094

**Sodium Azide** : Fisher Catalog #S0489100G

***C. elegans* strains: All strains were provided by the Caenorhabditis Genetics Center (CGC), which is funded by NIH Office of Research Infrastructure Programs (P40 OD010440).**

**EAK102 and EAK103 :** *C. elegans* models for Huntington's disease, in which versions of a fragment of the human Huntingtin protein tagged with YFP is expressed in the body wall muscles (Lee et al., 2017; Ung et al., 2020). EAK102 expresses the Huntingtin protein with the normal number of glutamine repeats (Q15), and EAK103 expresses the mutant Huntingtin protein with the aggregation-inducing number of glutamine repeats (Q128).

**N2 :** Bristol wildtype *C. elegans* strain.

**VC4126 :** Triple mutant with the interest of *cnnm-5* genetic nonsense mutation ( *gk5208* T->A) preventing ubiquitous expression.

**GMC101 :** Alzheimer's disease (AD) model strain, expresses human A-beta-1-42 peptide in body wall muscle cells that aggregates *in vivo* , paralysis induced by shifting to 25°C at L4 stage to cause accumulation.

**MAH215 :** Expression of autophagy protein LGG-1 with dual fluorescent tag (mCherry::GFP: *: lgg-1* ), used as a tool to measure autophagic flux (Chang et al., 2017).

**Table:** 

Strain	Genotype	Source
EAK102	*eeeIs1* [ unc-54 p::Htt513(Q15)::YFP:: unc-45 3'UTR]	CGC
EAK103	*eeeIs2* [ *unc-54* p::Htt513(Q128)::YFP:: *unc-45* 3'UTR]	CGC
N2	Bristol Wildtype	CGC
VC4126	Y39G10AR.15 ( *gk5206* ) ZC334.7 ( *gk5207* ) I; *cnnm-5* ( *gk5208* ) III. *gk5206* mutation T->A *gk5207* mutation C->T *gk5208* mutation T->A	CGC
GMC101	*dvIs100* [ *unc-54* p::A-beta-1-42:: *unc-54* 3'-UTR + *mtl-2* p::GFP]	CGC
MAH215	*sqIs11* [ *lgg-1* p::mCherry::GFP:: *lgg-1* + *rol-6* ]	CGC

## References

[R1] Chang Jessica T, Kumsta Caroline, Hellman Andrew B, Adams Linnea M, Hansen Malene (2017). Spatiotemporal regulation of autophagy during Caenorhabditis elegans aging. eLife.

[R2] Chen Yu Seby, Gehring Kalle (2023). New insights into the structure and function of CNNM proteins. The FEBS Journal.

[R3] Finkbeiner S. (2011). Huntington's Disease. Cold Spring Harbor Perspectives in Biology.

[R4] Giménez-Mascarell Paula, González-Recio Irene, Fernández-Rodríguez Cármen, Oyenarte Iker, Müller Dominik, Martínez-Chantar María Luz, Martínez-Cruz Luis Alfonso (2019). Current Structural Knowledge on the CNNM Family of Magnesium Transport Mediators. International Journal of Molecular Sciences.

[R5] Ishii Tasuku, Funato Yosuke, Hashizume Osamu, Yamazaki Daisuke, Hirata Yusuke, Nishiwaki Kiyoji, Kono Nozomu, Arai Hiroyuki, Miki Hiroaki (2016). Mg2+ Extrusion from Intestinal Epithelia by CNNM Proteins Is Essential for Gonadogenesis via AMPK-TORC1 Signaling in Caenorhabditis elegans. PLOS Genetics.

[R6] Lee Amy L., Ung Hailey M., Sands L. Paul, Kikis Elise A. (2017). A new Caenorhabditis elegans model of human huntingtin 513 aggregation and toxicity in body wall muscles. PLOS ONE.

[R7] McColl Gawain, Roberts Blaine R, Pukala Tara L, Kenche Vijaya B, Roberts Christine M, Link Christopher D, Ryan Timothy M, Masters Colin L, Barnham Kevin J, Bush Ashley I, Cherny Robert A (2012). Utility of an improved model of amyloid-beta (Aβ1-42) toxicity in Caenorhabditis elegans for drug screening for Alzheimer’s disease. Molecular Neurodegeneration.

[R8] Porta-de-la-Riva Montserrat, Fontrodona Laura, Villanueva Alberto, Cerón Julián (2012). Basic <em>Caenorhabditis elegans</em> Methods: Synchronization and Observation. Journal of Visualized Experiments.

[R9] Qu Wubin, Ren Changhong, Li Yuan, Shi Jinping, Zhang Jiye, Wang Xiaolei, Hang Xingyi, Lu Yiming, Zhao Dongsheng, Zhang Chenggang (2011). Reliability analysis of the Ahringer Caenorhabditis elegans RNAi feeding library: a guide for genome-wide screens. BMC Genomics.

[R10] Ravikumar Brinda, Vacher Coralie, Berger Zdenek, Davies Janet E, Luo Shouqing, Oroz Lourdes G, Scaravilli Francesco, Easton Douglas F, Duden Rainer, O'Kane Cahir J, Rubinsztein David C (2004). Inhibition of mTOR induces autophagy and reduces toxicity of polyglutamine expansions in fly and mouse models of Huntington disease. Nature Genetics.

[R11] Riddle, D. L., Blumenthal, T., Meyer, B. J., & Priess, J. R. (1997). *C. elegans II. 2nd edition. Section I, The Biological Model.* Cold Spring Harbor (NY): Cold Spring Harbor Laboratory Press. https://www.ncbi.nlm.nih.gov/books/NBK20086/

[R12] Saudou Frédéric, Humbert Sandrine (2016). The Biology of Huntingtin. Neuron.

[R13] Ung Hailey M., Hall Rhodes H. , Kikis Elise A. (2020). Chemical mutagenesis of Caenorhabditis elegans uncovers genetic modifiers of huntingtin protein aggregation.

